# Estimates of Workload Associated With Suicide Risk Alerts After Implementation of Risk-Prediction Model

**DOI:** 10.1001/jamanetworkopen.2020.21189

**Published:** 2020-10-21

**Authors:** Andrea H. Kline-Simon, Stacy Sterling, Kelly Young-Wolff, Gregory Simon, Yun Lu, Monique Does, Vincent Liu

**Affiliations:** 1 Division of Research, Kaiser Permanente Northern California, Oakland; 2 Kaiser Permanente Washington Health Research Institute, Seattle; 3 The Permanente Medical Group, Kaiser Permanente, Oakland, California

## Abstract

This cohort study attempts to validate the Mental Health Research Network suicide risk–prediction model and estimate associated workloads.

## Introduction

From 1999 to 2017, the US suicide rate increased by 35% across age, sex, and geographic groups.^1^ Suicide risk–prediction models using data from electronic health records provide a promising approach for identifying and assisting individuals at risk.^2^ The Mental Health Research Network (MHRN) developed highly discriminative suicide risk–prediction models using data from 20 million mental health care visits across 7 health systems.^3,4^ However, the clinical and operational requirements for implementing the models in practice are unknown. We sought to externally validate the MHRN risk model and provide clinical workload estimates for implementation.

## Methods

The Kaiser Permanente Northern California institutional review board approved this cohort study and waived the need for informed consent because the research involved no more than minimal risk to the participants, the research could not be practicably carried out without the waiver, and the waiver did not adversely affect the rights and welfare of the participants. As such, the study met the waiver criteria under 45 CFR 46.116(f). This report followed the Strengthening the Reporting of Observational Studies in Epidemiology (STROBE) reporting guideline for cohort studies. Kaiser Permanente Northern California is an integrated health care–delivery system serving 4.3 million members, 12% of whom are Medicaid beneficiaries and 58% of whom are members of racial/ethnic minority groups.^5^ We included all mental health encounters at Kaiser Permanente Northern California from October 1, 2016, to September 30, 2017. The MHRN suicide-attempt model used electronic health record measures, including demographic characteristics, Patient Health Questionnaire-9 item 9 scores,^6^ comorbidities, medications, mental health visits, and suicide attempts in the 5 years before the encounter date. Differences in data systems required minor modifications (ie, dropping census-derived information, combining race/ethnicity and insurance categories, and simplifying Patient Health Questionnaire-9 variables). We assessed discrimination of the MHRN model by examining suicide attempts within 90 days of encounters with an area under the receiver operating characteristic curve. To examine potential workload arising from risk alerts, we calculated the expected number of alerts at differing risk thresholds, ranging from the top 5% to the top 0.5% of scores. Analyses were conducted between January 2019 and March 2020. Stata/SE statistical software version 14.2 (StataCorp) and SAS statistical software version 9.4 (SAS Institute) were used for calculating summary statistics and area under the receiver operating characteristic curve.

## Results

**Figure 1.**
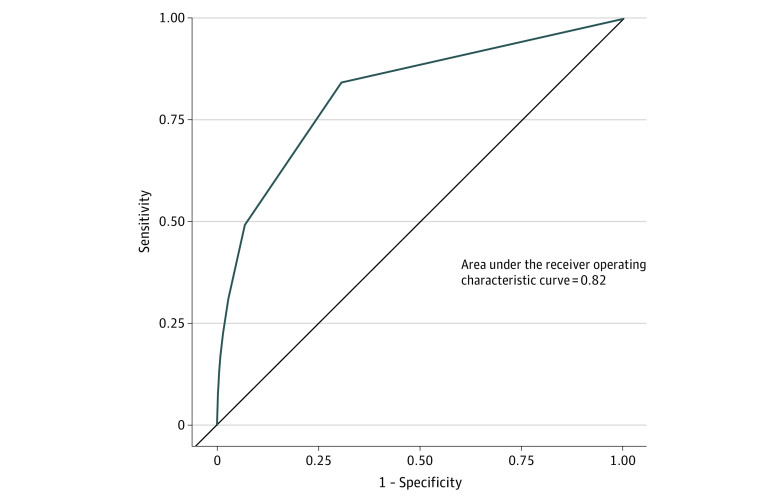
Model Discrimination for Kaiser Permanente Northern California Sample

**Figure 2.**
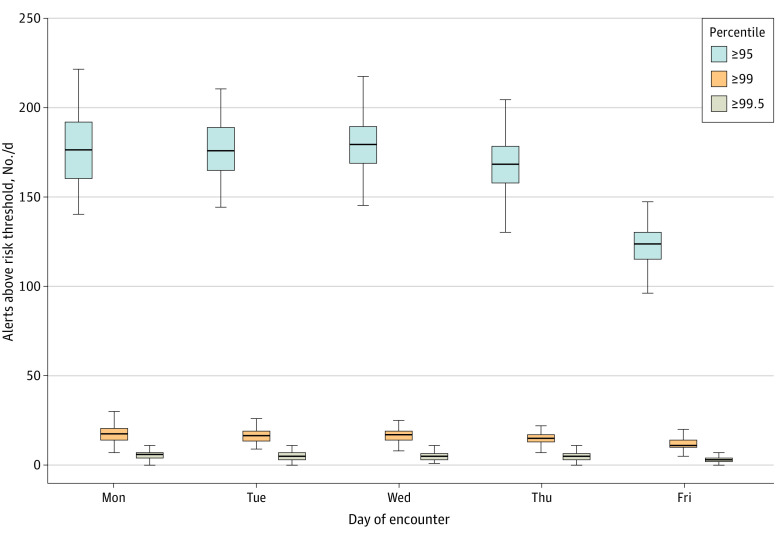
Estimated Daily Alerts

Over 1 year, we identified 1 408 683 mental health encounters (254 779 unique patients with mean [SD] age 40.7 [18.7] years, including 89 857 men [35.3%], 63 110 individuals who were Hispanic or Black [24.8%], and 35 267 individuals who had Medicare coverage in the previous year [13.8%]). Patients had a median (interquartile range [IQR]) 11 (5-24) visits, and 9054 patients attempted suicide within 90 days of a visit (0.6%). Model discrimination (area under the curve, 0.82; 95% CI, 0.81-0.82; Figure 1) was comparable to that found by Simon et al^3^ using the original MHRN sample (area under the curve, 0.851). The 95th percentile cut point had a sensitivity of 41.3% (95% CI, 39.5%-43.3%) and positive predictive value of 6.4% (95% CI, 6.2%-6.7%). The median number of daily risk alerts varied widely based on specified thresholds (Figure 2).

Excluding weekend mental health visits (1875 visits [0.1%]), the median [IQR] daily number of visits with suicide risk alerts across the region was 162 (117-182) visits for the 95th percentile and above, 14 (10-18) visits for the 99th percentile and above, and 4 (2-6) visits for the 99.5th percentile and above cutoffs. When limited to patients’ first visits through the end of the study period, daily median alerts were significantly lower, at 2 (0-4) visits for the 95th percentile and above cutoff and 0 visits for the 99th percentile and above and 99.5th percentile and above cutoffs.

## Discussion

The findings of this cohort study suggest that suicide risk models can accurately stratify risk but will generate additional workload for clinicians. The degree of added workload depends on the risk threshold selected, the strategy for responding to repeated alerts per patient, and the overlap of electronic alerts with risks already identified by treating clinicians. Selecting a risk threshold also depends on the relative importance of avoiding false-negative and false-positive errors. Without estimates for these factors, attempts to design a system to respond to alerts will be hampered. However, understanding alert characteristics alone is insufficient for developing these programs, as there remain many key effectiveness, clinical, operational, ethical, and legal questions regarding implementation of these programs .

This cohort study has several limitations. Findings may not generalize to all health care systems. Available electronic health record data did not include measures of relevant life events or patient-reported outcomes, except for data from the Patient Health Questionnaire-9. Additionally, the efficacy of interventions associated with suicide risk alerts remains uncertain.

This study adds to the evidence supporting the use of suicide risk-prediction models to augment traditional clinician assessment. The study’s new data further suggest that an appropriate alert threshold could limit the burden on clinicians.
